# Evaluation of Commercially Available Chikungunya Virus Immunoglobulin M Detection Assays

**DOI:** 10.4269/ajtmh.16-0013

**Published:** 2016-07-06

**Authors:** Barbara W. Johnson, Christin H. Goodman, Kimberly Holloway, P. Martinez de Salazar, Anne M. Valadere, Michael A. Drebot

**Affiliations:** ^1^ Diagnostic and Reference Laboratory, Arboviral Diseases Branch, Division of Vector-Borne Diseases, Centers for Disease Control and Prevention, Fort Collins, Colorado.; ^2^ Viral Zoonosis, National Microbiology Laboratory, Public Health Agency of Canada, Winnipeg, Canada.; ^3^ Caribbean Public Health Agency, Port of Spain, Trinidad and Tobago.

## Abstract

Commercial chikungunya virus (CHIKV)–specific IgM detection kits were evaluated at the Centers for Disease Control and Prevention (CDC), the Public Health Agency of Canada National Microbiology Laboratory, and the Caribbean Public Health Agency (CARPHA). The Euroimmun Anti-CHIKV IgM ELISA kit had ≥ 95% concordance with all three reference laboratory results. The limit of detection for low CHIK IgM+ samples, as measured by serial dilution of seven sera up to 1:12,800 ranged from 1:800 to 1:3,200. The Euroimmun IIFT kit evaluated at CDC and CARPHA performed well, but required more retesting of equivocal results. The InBios CHIKjj *Detect* MAC-ELISA had 100% and 98% concordance with CDC and CARPHA results, respectively, and had equal sensitivity to the CDC MAC-ELISA to 1:12,800 dilution in serially diluted samples. The Abcam Anti-CHIKV IgM ELISA had high performance at CARPHA, but at CDC, performance was inconsistent between lots. After replacement of the biotinylated IgM antibody controls with serum containing CHIKV-specific IgM and additional quality assurance/control measures, the Abcam kit was rereleased and reevaluated at CDC. The reformatted Abcam kit had 97% concordance with CDC results and limit of detection of 1:800 to 1:3,200. Two rapid tests and three other CHIKV MAC-ELISAs evaluated at CDC had low sensitivity, as the CDC CHIKV IgM in-house positive controls were below the level of detection. In conclusion, laboratories have options for CHIKV serological diagnosis using validated commercial kits.

## Introduction

Chikungunya virus (CHIKV) is an arthropod-borne alphavirus that causes acute epidemic polyarthralgia and febrile illness.^1^–^4^ Since 2004, CHIKV has reemerged in Africa, Asia, and Oceania, resulting in millions of human infections.^1^,^5^–^7^ In the fall of 2013, local transmission of CHIKV was reported on Saint Martin Island in the Caribbean.^8^,^9^ Since then the virus has disseminated rapidly throughout the Americas.^10^ In the United States, dengue (DEN) and CHIK became reportable diseases in January 2010 and January 2015, respectively.

Clinical illness is characterized by acute onset of joint pain, followed by myalgia, rash, and fever.^3^ Patients usually recover within a week, although some patients report long-term arthralgia. Dengue viruses (DENVs) co-circulate in many of the regions where CHIKV has emerged, and clinical symptoms between the two diseases are similar.^11^,^12^ Laboratory-based diagnosis is essential to differentiate CHIKV from DENV infections, as the clinical care of the patients may be different.^13^

The primary laboratory test used to diagnose CHIKV infection in serum collected within the first 5 days after onset is detection of CHIKV or viral RNA, after which the CHIKV-specific IgM antibody-capture enzyme-linked immunosorbent assay (MAC-ELISA) is a sensitive test.^14^ There are a variety of commercially available CHIKV IgM detection assays, but assessments of their performance have been limited.^15^–^20^ Comprehensive evaluations of the commercial assays were needed to harmonize results, assess assay performance, and provide recommendations for their use. The assessments were done in three laboratories: the U.S. Centers for Disease Control and Prevention (CDC) Arboviral Diseases Branch, the Public Health Agency of Canada National Microbiology Laboratory (NML), and the Caribbean Public Health Agency (CARPHA). All nine of the commercially available kits were evaluated at CDC with a panel of well-characterized archived diagnostic sera (Table 1 ). NML evaluated the Euroimmun Anti-CHIKV IgM ELISA with samples submitted for diagnostic testing from Canadian travelers who had recently visited CHIKV-endemic areas. Comparative testing with the Euroimmun, Abcam, and InBios CHIKV MAC-ELISA kits and the Euroimmun CHIK IgM indirect immunofluorescent test (IIFT) was done at CARPHA with selected samples from patients and controls from different CARPHA member states in the Caribbean, which had initially been tested at CDC.

## Materials and Methods

### CDC evaluation.

#### CDC samples.

A panel of serum samples without personal identifiers was selected from the CDC Arboviral Diseases Branch collection of archived diagnostic specimens. Reference testing had been performed at CDC according to the following algorithm. Samples that had been collected < 6 days after onset of illness were tested by real-time reverse transcription polymerase chain reaction (RT-PCR) using two sets of primers and probes designed by R. S. Lanciotti at CDC (Ref. 14 and CHIKV3855f, GAGCATACGGTTACGCAGATAG; CHIKV3957c, TACTGGTGATACATGGTGGTTTC + TGCTGGTGACACATGGTGGTTTC; CHIKV3886 FAM, ACGAGTAATCTGCGTACTGGGACGTA + ACGAGTCATCTGCGTATTGGGACGCA). A positive result with both sets of primers/probes was considered confirmatory, and the samples were not tested further. Those with negative results and samples collected ≥ 6 days after illness onset were tested by the CDC in-house MAC-ELISA.^21^ Samples were diluted 1:400 and run according to standard operating procedures (SOPs) in triplicate, against viral and normal antigens.^21^ Samples with positive and equivocal (EQ) ELISA results were confirmed by CHIKV 90% serum dilution endpoint plaque reduction neutralization assay (PRNT) in Vero cells using a 0.5% agarose double overlay, with 0.005% neutral red in the second overlay, applied after 2 days of incubation.^22^,^23^ CHIKV strain S27 was used as the challenge virus. The lower level of quantitation was 1:10 dilution. The CDC test results were considered CHIK IgM positive or negative. The original panel was composed of 92 samples that had all been tested by CDC CHIKV MAC-ELISA: 46 samples positive for CHIKV IgM from outbreaks of CHIKV Asian strain in Yap (2013) and the Caribbean (2014) and travelers to the Philippines; four pooled samples of CHIKV IgM in-house positive controls (IHPCs) with low, medium, and high positive-to-negative ratios (P/N) (CDC P/N approximately 4.5, 10, and 20, respectively) from outbreaks of CHKV East/Central/South African strain in Comoros (2005) and India (2006); 36 samples negative for CHIKV and other arbovirus IgM, from patients with fever from Thailand, Yap, and the Caribbean; and six IgM IHPCs for DEN (*N* = 2), o'nyong-nyong (ONN), Mayaro (MAY), Venezuelan equine encephalitis (VEE), and North American eastern equine encephalitis (EEE) viruses. The MAYV and ONNV IgM IHPCs had positive results by CDC CHIKV MAC-ELISA and were considered CDC CHIK IgM+ (cross-reactive), but had been confirmed as CHIK negative by PRNT. Samples were coded CHIKV IgM+ or CHIKV IgM−. In the CDC MAC-ELISA, the calculated P/N is not a quantitative unit: P/N < 2 is negative, P/N 2–3 is EQ, and P/N > 3 is positive.^21^ However, samples were chosen so that there was a range of P/N ratios represented: approximately 30% P/N 3–5 (low positives), 40% with P/N 5–10, and 30% with P/N > 10. The number of days from onset of illness to sample collection ranged from 2 to 33. Samples with similar results were added as volumes were depleted. All samples were labeled using a code that was devoid of personal identifiers. Because of this testing algorithm, there were no samples in the panel that had positive results by real-time RT-PCR, which had been subsequently tested by the CDC CHIK MAC-ELISA.

#### CDC test methods.

The samples in the reference panel had previously been tested in the CDC diagnostic laboratory, and CDC results were considered the reference standard. The CDC uses the same ELISA format for testing all arboviruses, with previously optimized sample dilutions of 1:400.^24^ Although the sensitivity and specificity of the CDC MAC-ELISA has not been determined for each arbovirus, the assay was shown to have sensitivity and specificity of 91% and 90%, respectively, for West Nile virus when samples with EQ results were confirmed by PRNT.^25^,^26^ All kit manufacturers or distributors were contacted by CDC about the evaluations, and kits used in the evaluations were either donated by the suppliers or purchased by CDC. Results of the evaluations were reported to the manufacturer of each kit, and all manufacturers were afforded the opportunity to modify the kits and resubmit them for reevaluation. All kits were stored as prescribed by the manufacturer. The samples were diluted and tests were run according to the manufacturers' instructions in the kit insert. Because reference panel sample volumes were limited, each kit was initially tested with a subset of 8–10 samples which included a CHIKV IHPC and normal (CHIKV IgM− control, IHNC) serum. If the results of the IHPC and IHNC were correct, the rest of the samples in the panel were tested. The kit results were classified as CHIKV IgM+, CHIKV IgM−, or EQ according to the cutoff values provided by the manufacturer. EQ results were retested. Results that remained EQ after retesting were coded as negative. Replicate testing was not routinely undertaken, as this would not typically be done in the diagnostic laboratory, with the following exceptions. In the Abcam kit standard protocol, antigen was incubated in the plate for 30 minutes at room temperature. In the troubleshooting section of the instructions, there was a suggestion to incubate the antigen overnight at 4°C if there was low signal. Samples were tested by both methods. Samples were also retested with different lots of the Abcam and Euroimmun ELISA kits. In addition, after initial testing by the Euroimmun, Abcam, and InBios kits, samples with discordant results were retested by both the CHIKV CDC MAC-ELISA and kit, and any samples with CDC results that changed after retesting were excluded from the evaluation.

To test sensitivities of the kits, one CDC CHIKV IHPC and six CDC CHIKV IgM+ samples were diluted 2-fold to 1:12,800 in sample dilution buffer and tested. According to the instructions in the Euroimmun ELISA kit, samples were incubated for 10 minutes in the sample dilution buffer, as it contains antihuman IgG antibodies that bind the IgG in the sample. To control for differences in results due to the incubation step, samples tested by the Euroimmun ELISA were prepared by diluting before and after incubation in the sample dilution buffer, and both dilution series were tested.

### NML evaluation.

#### NML samples.

A total of 247 serum samples were selected for the Euroimmun IgM kit evaluation; 100 previously confirmed CHIKV IgM+ diagnostic serum samples, 99 diagnostic samples determined to be CHIK IgM− but harboring pathogen-specific antibodies (IgM and/or IgG) against a number of other human pathogens, and 48 clinical samples that had been shown to be CHIKV IgM− and with no recorded positive serological results for other infectious agents (Tables 2 and 3 ). Of the 100 confirmed CHIKV IgM+ serum samples, 63 were collected from suspected cases of CHIK with travel history to the Caribbean and other endemic areas in the Americas; two each had travel history to Asia, Africa, and Oceania. However, 31 CHIKV IgM+ sera cases had no travel history information (Table 2).

Serum samples selected for Euroimmun Anti-CHIKV ELISA kit evaluations were previously tested by an in-house CHIKV MAC-ELISA based on the CDC MAC-ELISA method described above, with the exceptions that samples were tested in duplicate and the assay used tissue culture–derived antigen produced at NML.^21^ Samples that generated CHIKV IgM+ results were further tested by PRNT and/or real-time RT-PCR and/or, for the purposes of this study, hemagglutination inhibition assay (HAI) to confirm clinical cases (Table 2). The PRNT was performed as the gold standard confirmatory assay essentially as described above using CHIK strain S27 as the challenge virus, with the exception that the second overlay was applied at approximately 3 days.^22^

The real-time RT-PCR assay was added to confirm any samples that were CHIKV IgM+ but had a negative result in the PRNT as well as to determine the number of CHIKV viremic individuals returning to Canada. In the real-time RT-PCR testing, viral RNA was extracted from clinical samples using the Qiagen QIAamp Viral RNA mini kit (Hilden, Germany) according to manufacturer's protocol. Amplification was performed on a Vii A 7 (Applied Biosystems, Foster City, CA) using the TaqMan Fast Virus 1 Step Master Mix (Life Technologies, Carlsbad, CA) according to manufacturer's protocol. Viral extracts were tested by two singleplex reactions using two sets of primers and probes, one described by Pongsiri and others^27^ and the other designed by R. S. Lanciotti at CDC (see above section “CDC samples”). Samples that generated Ct values of < 38 in both singleplex reactions were classified as reactive. For the purposes of this project, an HAI assay was used for supplemental confirmatory testing using suckling mouse brain antigen produced at NML according to the protocol of Beaty and others.^22^,^28^

The 100 NML CHIKV IgM+ samples exhibited a range of P/N values: 21 samples exhibited low positive values (P/N: 3–4.9), 28 samples demonstrated moderate positive values (P/N: 5–9.9), and 51 samples generated high P/N values (P/N ≥ 10).

#### NML test methods.

Samples were tested twice in separate runs in the Euroimmun IgM ELISA as per manufacturer's instructions. If there was disparity between replicate results, samples were run a third time and the two concordant results were averaged and used for data analysis.

### CARPHA evaluation.

#### CARPHA samples.

Samples used in the panel were representatives from CARPHA-associated islands. Aliquots of samples had been previously referred to CDC during the outbreak of CHIK in the Caribbean in early 2014, as there were no characterized in-house controls that could be used for diagnostic testing. CDC results were considered the reference standard for the evaluation. All samples were labeled using a code that was devoid of personal identifiers. In addition, a set of samples predating the outbreak was used for assessing cross-reactivity (*N* = 12), which were positive at CARPHA for DEN (*N* = 4), cytomegalovirus (CMV) (*N* = 2), Epstein–Barr virus (EBV) (*N* = 2), hepatitis A virus (HAV) (*N* = 2), and leptospirosis (*N* = 2); as these samples were not tested at CDC, results were not included for calculation of accuracy, sensitivity, and specificity.

#### CARPHA test methods.

Abcam, InBios, and Euroimmun ELISA kits and the Euroimmun IIFA (IgM) kit were stored and run as prescribed by manufacturer's instructions with the exception of overnight incubation with Abcam antigen for approximately 16–18 hours at 4°C. Because of limited available quantity of sera, subsets of 36, 27, 21, and 26 samples considered CHIKV IgM+ by CDC and 10, 14, 12, and 10 samples considered IgM− by CDC were used for evaluating the Abcam, InBios, Euroimmun IIFA, and Euroimmun ELISA kits, respectively. To evaluate within-run and between-run variances, a set of samples was tested in duplicate in the same plate as well as tested in a second run. Furthermore, an alternate kit lot number was used to evaluate Abcam and InBios between-lot variance.

#### Statistical methods.

For the purposes of this evaluation, test results were considered in two categories, CHIKV IgM+ and CHIKV IgM−. EQ results from test kits were coded negative for analysis. Sensitivity was defined as the proportion of samples with a reference standard result of CHIKV IgM+ that had a CHIKV IgM+ result in the test kit. Specificity was defined as the percentage of reference standard CHIKV IgM− results that had CHIKV IgM− results in the kit. Accuracy was defined as the agreement of results between the evaluated kit and the reference standard assay. The 95% confidence intervals (CIs) for proportions were calculated according to the efficient-score method, corrected for continuity based on the procedure outlined by Wilson.^29^,^30^

## Results

The Euroimmun ELISA was evaluated at CDC, NML, and CARPHA; the Abcam and InBios ELISA kits and the Euroimmun IIFT were evaluated at CDC and CARPHA; and the CTK, Genway, and SD Diagnostics ELISA kits and the CTK and SD BIOLINE CHIKV IgM rapid tests were evaluated only at CDC. Characteristics of the sample sizes and specimen type and the test results are shown in the corresponding tables: CDC (Table 4 ), NML (Tables 2 and 3), and CARPHA (Table 5 ). The summary results of the evaluations from all three laboratories for the four kits with the highest performance are shown in Table 6.

### CDC evaluations.

Nine CHIKV IgM detection assays were assessed at CDC with a panel of serum specimens that had previously been submitted to and tested at the CDC arbovirus diagnostic laboratory (Table 1). CDC results and final interpretations were considered the reference standard. Samples were tested by the commercial IgM detection assays listed according to manufacturers' instructions. Test runs were considered valid or invalid according to the criteria in the SOP; only valid test results are reported here. A CDC CHIKV IHPC was included in every run. At the end of the evaluation, samples with discordant results were retested by the CDC CHIKV MAC-ELISA. If the CDC results changed between initial diagnostic testing and retesting, the sample was excluded from the evaluation panel.

#### ELISA kits.

**Figure 1.**
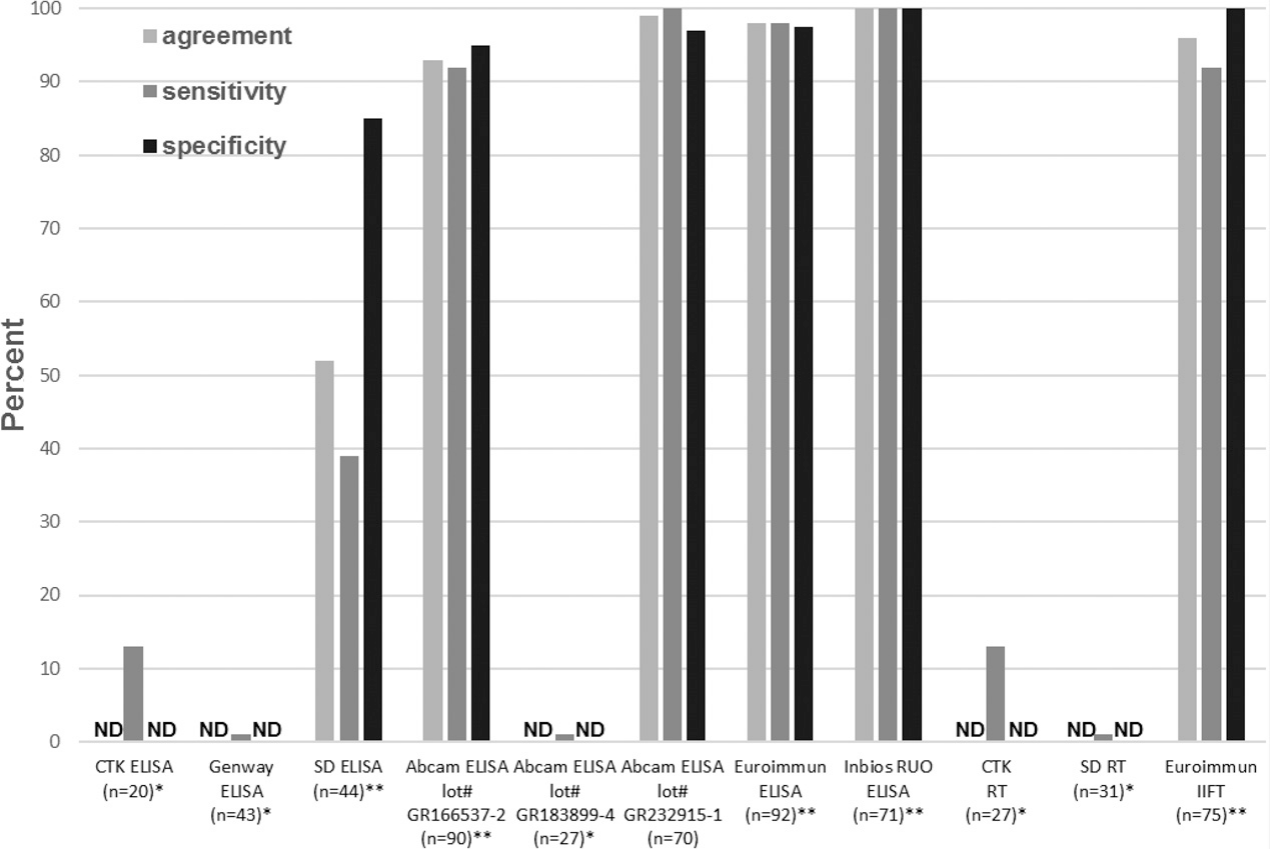
Performance of CHIKV IgM detection assays compared with CDC results as the reference standard. *CDC CHIKV IHPC had negative result in test. **CDC CHIKV IHPC had positive result in test. CDC = Centers for Disease Control and Prevention; CHIKV = chikungunya virus; IHPC = in-house positive control; ND = not done; RT = rapid test.

Of the samples, 20 were tested by the CTK ELISA. Of the 16 CDC CHIKV IgM+ samples, two had a positive result in the CTK ELISA; the CDC CHIKV IHPC had a negative result (Table 4, Figure 1

). The Genway ELISA was initially evaluated with 43 samples, 27 CDC IgM+ and 16 CDC IgM−. None of the CDC CHIKV IgM+ samples, including the CDC CHIKV IHPC, tested in two replicates, had a positive result in the Genway ELISA kit (Table 4, Figure 1). The SD ELISA was evaluated with 44 samples. Of the 31 CDC CHIKV IgM+ samples, 12 were positive by the SD ELISA, including the CDC CHIKV IHPC; two of the 13 CDC CHIKV IgM− samples were positive by the SD ELISA. These three assays had sensitivities of < 50% and therefore were not further evaluated.

**Figure 2.**
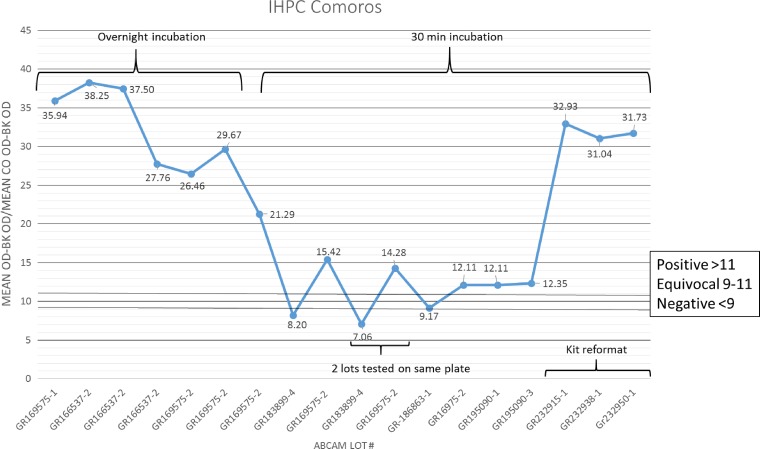
Abcam CHIK MAC-ELISA performance assessment with CDC CHIKV IHPC, by lot number. CDC = Centers for Disease Control and Prevention; CHIKV = chikungunya virus; IHPC = in-house positive control.

Results of the Abcam ELISA with a subset of samples were > 90% concordant with CDC results (data not shown). The rest of the panel was then tested in duplicate in two runs according to the SOP, with an antigen incubation time of 30 minutes at room temperature, and alternately, overnight at 4°C (Table 4). Of the 48 CDC CHIK IgM+ samples, 44 and 47 were positive by the Abcam test with 30 minutes and overnight antigen incubations, resulting in sensitivities of 92% (95% CI: 79–97%) and 98% (95% CI: 88–100%), respectively. Performance indicators of the test with 30 minutes antigen incubation are shown in Figure 1 (lot no. GR166537-2). Of the 42 CDC CHIKV IgM− samples, three and one had EQ results (coded as negative) in the Abcam assay incubated 30 minutes or overnight, respectively, and had specificities of 95% (95% CI: 83–99%) and 98% (95% CI: 86–100%), respectively. One sample with an EQ result in the CDC ELISA with no neutralizing antibody titer was classified as CDC CHIKV IgM−. This sample had a positive result in the Abcam assay under both antigen incubation conditions. The CDC ONNV IHPC had a negative result in the Abcam assay; the MAYV IHPC was not included in this initial testing. The DENV IHPC with the CDC CHIKV IgM+ result had a negative and positive result in the Abcam test with the antigen incubated 30 minutes and overnight, respectively. Accuracy of the Abcam kit, therefore, was 93% (95% CI: 86–97%) with 30 minutes antigen incubation and 98% (95% CI: 91–100%) with overnight antigen incubation. However, approximately 4 months later, an Abcam kit with a different lot number (GR183899-4) was purchased and none of the CDC CHIKV IHPCs or IgM+ samples tested with the new lot kit had a positive result (Figures 1 and 2

), including samples that previously had been tested with the Abcam kit and had correct results. To investigate the cause of the difference in performance between the two kit lots, reagents from the first lots of kits received were swapped one by one with reagents from the newest lot. The assay worked as expected when the biotinylated antibody from the new lot was replaced with biotinylated antibody from the old lot, indicating lot-to-lot variability in the activity of the biotinylated antibody. It was notable that, throughout all these runs, the optical densities (ODs) of the kit positive and cutoff controls were in the valid range, despite the negative result for the IHPCs with the new lot kits and even if no antigen or biotinylated antibody was added to the control wells (lot no. GR183899-4; Figure 2). Abcam was notified of the poor performance of the newer lot kits, and the nonspecific reactivity of the controls. As a result, the kit components were modified as follows. The kit cutoff and positive controls were replaced with CHIKV IgM−specific human serum, and the concentration of the biotinylated antibody was adjusted to increase sensitivity with a validation panel of low positive samples with ODs just above the cutoff. Because of the substantial changes made to the kit components, the performance indicators of the initial evaluation described above were invalidated, and the revised kits needed to be reevaluated. Abcam sent three lots of the revised kits to CDC, one (lot no. GR232915-1) of which was used to test a panel of 70 samples (Table 4, Figures 1 and 2). The other two lots were evaluated with a subset of samples and had equivalent results. Coding the EQ result as NEG, the revised Abcam ELISA had sensitivity of 100% (95% CI: 88–100%), specificity of 97% (95% CI: 83–100%), and overall agreement of 99% (95% CI: 91–100%). The MAYV and ONNV IHPC had positive results in both the CDC and Abcam tests.

Results of the Euroimmun ELISA with a subset of samples were 100% concordant with CDC results (data not shown); therefore the rest of the panel of 92 specimens was tested in two runs. Of the 52 CDC CHIKV IgM+ samples, 51 were positive by Euroimmun ELISA (sensitivity 98%; 95% CI: 88–100%); 38 of the 40 CDC CHIKV IgM− samples had a negative result and one had a EQ result (coded as negative) in the Euroimmun ELISA (specificity 97.5%; 95% CI: 85–100%) (Table 4, Figure 1). Accuracy of the Euroimmun kit with CDC results was 98% (95% CI: 92–100%). The MAYV and ONNV IHPC had positive results, similar to the CDC results. A subset of samples was retested with a Euroimmun kit from a different lot, and the results were equivalent (data not shown).

The InBios CHIK MAC-ELISA was evaluated with 71 samples, as there was not sufficient volume remaining to test the complete original panel (Table 4, Figure 1). The results were 100% concordant (95% CI: 94–100%) with CDC results, including positive results with the ONNV and MAYV IHPCs, and therefore had 100% sensitivity and specificity (95% CI: 88–100%).

#### Rapid tests.

The CTK rapid test was evaluated with a subset of 27 samples in two runs. Of the 23 CDC CHIKV IgM+ samples tested, three were positive by the CTK rapid test, but the CDC CHIKV IHPC had a negative result (Table 4, Figure 1). Thirty-one samples were tested in the SD rapid test in two runs. None of the 24 CDC CHIKV IgM+ samples, including the CDC CHIKV IHPC, had a positive result in the SD rapid test (Table 4, Figure 1). Neither of these two rapid tests was evaluated further.

#### IFA.

A subset of 16 samples initially was tested with the Euroimmun CHIKV IgM IIFT; results of 15 samples had concordant results. The rest of the panel of 75 samples was tested in two sequential runs. Of the 44 samples tested in the first run, eight samples with EQ (*N* = 4), weak positive, or invalid (*N* = 4) results from the first run were retested in the second run. After the second run, six samples needed retesting, three with EQ results and three CDC CHIKV IgM+ samples that had discordant results between the first and second run. Because there was insufficient volume for retesting, the EQ samples were not retested per the testing algorithm and coded as negative. Of the 37 CDC CHIKV IgM+ samples tested, 34 had positive results in the Euroimmun IIFT (sensitivity 92%; 95% CI: 77–98%) (Table 4, Figure 1). Three of the 38 CDC CHIKV IgM− samples that had EQ results were coded as negative, resulting in 100% specificity (95% CI: 89–100%). Overall accuracy of the Euroimmun IIFT kit was 96% (95% CI: 88–99%).

#### Comparison of performance between the CDC, Euroimmun, InBios, and Abcam ELISA.

Kit evaluations were done sequentially, and because of the limited sample volume and retesting needed, all kits were not evaluated with identical samples. To compare performance between the kits, the results of 68 samples (35 CDC CHIKV IgM+, 33 CHIKV IgM−) that had been tested in the Euroimmun, InBios, and revised Abcam ELISAs were used (Table 6). Results were 100% concordant between the CDC and the InBios ELISA and 99% concordant between the CDC and the revised Abcam and the Euroimmun ELISAs (Table 6).

#### Serum titration.

**Figure 3.**
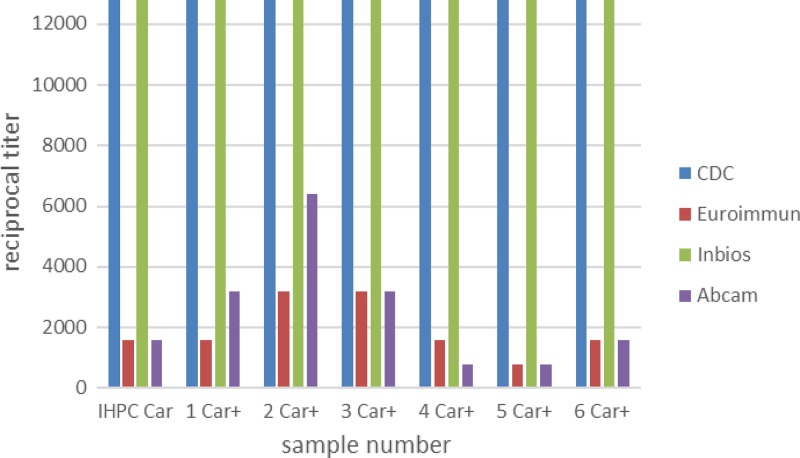
Comparison of sensitivity by serum titration. Chikungunya virus (CHIKV) IgM+ samples from the Caribbean were diluted 2-fold to 1:12,800 in sample dilution buffer. The revised Abcam kit with CHIKV-specific IgM serum controls was used for the evaluation.

The CDC CHIKV IHPC and six CDC CHIKV IgM+ samples from the Caribbean were diluted 2-fold from 1:100 to 1:12,800 and tested in the CDC, Euroimmun, InBios, and revised Abcam ELISAs. Sensitivities of the kits compared with the CDC MAC-ELISA are shown in Figure 3

. CHIKV IgM was detectable by the CDC and InBios assays to the highest dilution, whereas the endpoint of detection ranged from 1:800 in sample 5 in both the Euroimmun and Abcam assays, to 1:3,200 and 1:6,400, respectively, in sample 2.

### NML evaluations.

Of the 100 samples that tested IgM+ in the NML in-house CHIKV MAC-ELISA, 94 generated positive IgM results on the Euroimmun EIA (Table 2). Four samples had EQ results in the Euroimmun ELISA, of which three had been confirmed by CHIK real-time RT-PCR and one by PRNT. Two CHIKV IgM+ samples, which had been confirmed by real-time RT-PCR, had negative results in the Euroimmun ELISA. Among the 147 CHIKV IgM− samples tested, two parvovirus IgM+ and four *Anaplasma* IgG+ samples generated Euroimmun IgM+ results (Table 3). Overall the Euroimmun assay had 95% accuracy (95% CI: 91–97%), 94% sensitivity (95% CI: 87–98%), and 96% specificity (CI: 91–98%) at NML (Table 6).

### CARPHA evaluations.

Four CHIKV IgM detection assays were evaluated: the Abcam, Euroimmun, and InBios MAC-ELISAs and the Euroimmun IIFT (Table 5). Results of the testing at CDC were considered the reference standard. Accuracy of the Euroimmun ELISA (*N* = 36) was 100% (95% CI: 88–100%), with 100% sensitivity (26/26; 95% CI: 84–100%) and specificity (10/10; 95% CI: 66–100%). Within-run variance was 100% concordant (25/25), although a slight decrease in OD was observed for samples placed in later wells. Between-run variance (different plates with similar lot number) was 100% concordant (8/8). In the second run, the OD increased in most of the samples, although there was no change in the overall results. The only difference between the runs was an approximately 2 minute longer incubation period (from a total of 1 hour) in the second run.

Evaluation of Euroimmun IIFT for IgM detection (*N* = 33) showed 97% accuracy (95% CI: 82–100%), between CDC results and results achieved at CARPHA. Sensitivity was 100% (95% CI: 81–100%) and specificity was 92% (95% CI: 60–100%). Discordance occurred with false-positive results for one CDC IgM− sample. Evaluation of between-run and within-run variances showed 100% concordance (33/33). Because the IIFT requires a person to score the result based on their observations of the fluorescence of the cells under the microscope, a between-reader variance was evaluated. Results from an experienced reader were 100% concordant with CDC results (45/45), whereas results by a reader with no previous experience in IFA were 91% (41/45) concordant. Of note was the non-florescent green background seen with some samples, which could prove a difficulty for users new to IFA. This background was minimized with the use of an Evans blue counter stain commonly used at CARPHA with IFA. Slides originally washed with water distilled at CARPHA showed some background; however, this water had a pH of < 6.2. After replacement with commercially distilled water (pH 7.0), the background was reduced.

The Abcam ELISA kit had 100% accuracy (*N* = 46; 95% CI: 90–100%), sensitivity (36/36; 95% CI: 88–100%), and specificity (10/10; 95% CI: 66–100%) compared with CDC results. Within-run variance was 96% (24/25), and a general decrease in OD for samples placed in later wells was observed. Between-run variance evaluated by three runs was 100% concordant (19/19). Testing was performed with an approximate 16–18 hours overnight antigen incubation. The second run incubation was half an hour longer than first and third run and as expected, lead to a general increase in OD. Overall concordance of between-lot variance was 94% (16/17), as one sample was negative with one lot and inconclusive with a second lot. Moreover, the average ODs with the second lot were lower than with the first. An increase in the OD value of the blank well with the second lot was noted. However, raw OD values were also lower. Modifications were made to the Abcam kit after the CARPHA evaluation; these results are not valid for the kit in its current format and therefore are not included in summary (Table 6).

Overall concordance of the InBios kits (*N* = 41) with CDC results was 98% (95% CI: 86–100%), with 100% (27/27) sensitivity (95% CI: 84–100%) and 93% (13/14) specificity (95% CI: 64–100%). Within-run variance was 100% concordant (40/40) with overall OD correlation between wells > 0.998. Between-run variances with the same kit lot evaluated three times were 100% concordant (*N* = 40). Between-lot variances were found to be 98% concordant (46/47). Overall OD values with the second lot were slightly lower than the first lot.

The Euroimmun, InBios and Abcam ELISA kits showed no cross-reactivity with sera positive for anti-DEN, anti-CMV, anti-EBV, anti-HAV, and anti-*Leptospira* IgM antibodies. One sample positive for leptospirosis also had a positive result in the Euroimmun IIFT.

## Discussion

CHIKV, which reemerged in Asia and Oceania beginning in 2004 and emerged in the Americas in 2013, has caused large outbreaks, resulting in large numbers of serological samples submitted to diagnostic laboratories for testing (http://www.paho.org/hq/index.php?option=com_topics&view=article&id=343&Itemid=40931). The projected expansion and endemicity of CHIKV is likely, and laboratories will need to build and maintain high-volume diagnostic testing capacity in the future. Validated and reliable commercial CHIKV diagnostic assays are essential for these laboratories. CHIKV IgM detection assays are available commercially in various formats, including plate-based ELISAs, IIFT slides, and rapid tests. Assessments of these kits were conducted in three different diagnostic laboratory settings.

The CDC arbovirus diagnostic laboratory receives samples for reference diagnostic testing from outbreaks and from U.S. travelers to regions where CHIKV is circulating. Thus the evaluation panel was composed of confirmed, archived diagnostic samples. At NML, the Health Agency of Canada Arbovirus Reference and Diagnostic Laboratory, requests for CHIK testing in travelers has gone from 50 to 200 annually to 1,926 in 2014 and > 2,000 in the first 6 months of 2015. Most of the samples did not have accompanying information about the dates of illness onset or sample collection, or in some cases, travel history. The Euroimmun ELISA was evaluated with samples previously tested with NML in-house assays and which were predominately collected from individuals who had traveled to the Caribbean. In the NML testing algorithm, all samples were first tested by MAC-ELISA. Samples with IgM+ results were confirmed by a combination of real-time RT-PCR, PRNT, and for the purposes of this study, HAI. Interestingly, of the 100 CHIKV IgM+ samples, CHIKV RNA was also detected in 16 samples by real-time RT-PCR. Unfortunately, without information on the timing of the sample collection, no conclusions can be made about duration of viremia and immune response.

The third part of the evaluations were conducted at CARPHA, which is in the consortium of 26 member states of the English-speaking Caribbean islands and Haiti, Belize, Surinam, Guyana, Aruba, Curacao, Sint Maarten, and the BES islands. In late 2013, CARPHA began to receive specimens from the British Virgin Islands and Dominica for CHIKV diagnostic testing. Sample aliquots were initially sent to CDC for testing until CARPHA was able to implement testing. By the end of 2014, CARPHA had received approximately 4,500 samples from 19 CARPHA member states for CHIKV testing. Of the 3,000 samples tested, 54% were CHIKV positive. Initially, CDC ELISA reagents were supplied to CARPHA, but like other laboratories in the region, CARPHA will likely receive a large number of samples for the near future and will need to use a validated commercial assay to test them. A set of samples with results reported from CDC were selected to evaluate the Euroimmun, InBios, and Abcam ELISAs and Euroimmun IIFT kits at CARPHA. This provided an opportunity to assess the performance of the kits in a separate laboratory from CDC, where the initial testing had been done.

The Euroimmun ELISA was the only assay evaluated in all three laboratories. Accuracy was high, at 99%, 95%, and 100% for samples tested at CDC, NML, and CARPHA, respectively (Table 6). Sensitivity of the Euroimmun ELISA for low positive samples was measured by serially diluting seven CHIKV IgM+ samples from 1:100 to 12,800 (Figure 3). Compared with CDC testing, in which CHIKV IgM was detectable in all samples up to the final dilution, the endpoint of detection in the Euroimmun ELISA kit ranged from 1:800 to 1:3,200. At CARPHA, results of replicate testing to measure within-run and between-run variances were 100% concordant with CDC results, with some decrease in OD observed for samples located in later wells.

The Abcam Anti-CHIKV IgM human ELISA kit was evaluated at CDC and CARPHA. Two lots evaluated at CARPHA had 100% concordance with CDC results, and low variance between and within runs. At CDC the performance of the Abcam kits varied from lot to lot. Three lots had high sensitivity, whereas the sensitivity was so low in two lots that the IHPC did not have a positive result in the test, which invalidated the initial evaluation (Figure 2). It was notable that the test was still valid according to the criteria. By switching out the new lot reagents with old lot reagents one at a time, the problem was identified as low activity of the biotinylated antibody in the new lot. The kit positive control (PC) was within the acceptable range in all runs, leading to the conclusion that the PC was a biotinylated IgM, which reacted directly with the streptavidin, but which did not contain CHIKV-specific IgM. CDC reported the problem to Abcam, which subsequently contacted the original equipment manufacturer. The components of the kit were modified so that the biotinylated IgM+ and cutoff controls were replaced with serum with CHIKV-reactive IgM. These controls will now only give a positive signal if all the components of the sandwich ELISA are working properly. Second, quality control (QC) of the kits now includes low-positive serum samples in the evaluation panel; sensitivity of the kit has to be high enough to detect CHIKV IgM in these low-positive serum samples for the lot to be validated. In addition, the suppliers will notify the distributor of any changes made to the controls or QC process. These changes will allow for better oversight of the quality of the kits as well as set more stringent guidelines for the QC process (T von Will, Abcam, personal communication). Because the kit components were modified, the initial CDC Abcam kit evaluation and the CARPHA evaluation were no longer valid, and the reformatted kit needed to be reevaluated. Three lots of the kits containing the CHIKV-specific IgM serum controls were received and evaluated at CDC and had 99% concordance (Tables 4 and 6). In the serially diluted samples, the endpoint of detection in the Abcam ELISA kit ranged from 1:800 to 1:6,400. It is important to note that these results pertain only to the kit format with the CHIKV-specific IgM serum controls; any further modifications to the kit will invalidate these results and require reevaluation.

The InBios CHIKjj *Detect* MAC-ELISA Kit, evaluated at CDC and CARPHA, had high performance, with 100% and 98% accuracy, respectively. In the serially diluted samples, the InBios ELISA had the highest sensitivity of all the kits and equal to that of the CDC MAC-ELISA. Results were highly concordant with CDC results in replicate testing at CARPHA to determine within-run, between-run, and between-lot variance testing, at 100%, 100%, and 98% concordance, respectively.

The Euroimmun IIFT kit, evaluated at CDC and CARPHA, had high performance (accuracy 96% and 97%, respectively) but required more retesting of EQ results, due to background fluorescence from uninfected cells, which could be reduced somewhat by counterstaining. The proficiency of the technician also affected the interpretation of results and thus concordance, as side-by-side testing conducted at CARPHA with experienced and unexperienced readers showed.

Rapid tests are attractive because they are “low tech” as they do not need specialized, expensive equipment and do not require trained, experienced laboratory technicians to run and interpret the test. They can be performed in a clinical setting, or at the point of care, so that there is no need to ship the sample to a laboratory. However, as shown in previous studies and in this evaluation of the two lateral flow assays with sensitivities < 50%, rapid tests often lack sensitivity.^15^,^31^,^32^ In addition, a true negative cannot be distinguished from a false negative by the validity criteria, because there is no serum positive control; the assay control only confirms that the reagents have been applied and the buffer has moved across the strip and over the detector correctly. Because of the poor performance of the two rapid tests evaluated at CDC, their use is not recommended, despite their user-friendly format. The formats of the CTK and SD CHIKV MAC-ELISA kits were similar to other assays but had poor performance compared with the CDC MAC-ELISA. Both of these kits lacked the sensitivity to detect IgM in samples with low, medium, or high P/N values at CDC.

Commercial manufacturers produce MAC-ELISA kits for a variety of pathogens. Optimization of the assay for a new pathogen can be streamlined if most of the reagents remain the same and are previously standardized, as switching out reagents may affect performance significantly. In the Abcam kit, by using a biotinylated IgM PC, only the viral antigen needed to be changed, which simplified the optimization process. However, the validity criteria only measured the reactivity of the IgM PC with the anti-IgM Mab coated on the plate, not CHIKV-specific IgM reactivity with the CHIKV antigen. Use of a virus-specific IgM PC in the kit to measure validity is essential, as detection of the poor performance of particular lots of the Abcam ELISA at CDC, which had passed quality assurance/QC at the manufacturers, demonstrated.

The lot-to-lot variability of the Abcam kit also highlights the importance of using internal controls at the end-user laboratory. In the Abcam kit evaluation at CDC, without the inclusion of an IHPC in every run, the invalid runs would have been considered valid and the true CHIKV IgM+ samples would have had false-negative results. ELISAs are biological assays and many factors can have an impact on the accuracy of the assay. Internal controls help to monitor consistency and detect factors that cause error in testing in addition to the diagnostic test, such as the equipment, personnel, and environment. Including an IHPC in each run and documenting and charting the OD of the IHPC is an essential component of laboratory QC to assure that the laboratory results are accurate and the reproducibility of the kit performance is reliable.

Specificity was assessed at all three laboratories with specimens containing IgM to a variety of pathogens or no pathogen-specific IgM. As expected, there was very little nonspecific reactivity to most of these specimens. CHIKV is an alphavirus, and inclusion of alphavirus VEEV, EEEV, ONNV, and MAYV IgM PC serum at CDC and Ross River virus IgM at NML measured the CHIKV IgM specificity, or alphavirus cross-reactivity, in the assays. VEEV and EEEV IgM PC samples had negative results in the CDC MAC-ELISA and in the four kits. Similarly, the three Ross River virus IgM+ samples included in the NML panel did not react in the Euroimmun ELISA. MAYV and ONNV IgMs cross-reacted in the CDC CHIKV MAC-ELISA and showed similar reactivity in the Euroimmun, InBios, and Abcam ELISA kits (Table 4). Thus, the kits had equal specificity to the reference tests to VEEV, EEEV, and Ross River virus IgM and had lack of specificity equal to that of the CDC CHIKV MAC-ELISA to MAYV and ONNV IgM. MAYV was first identified on the Island of Trinidad and is known to circulate in tropical Americas.^33^ Although large outbreaks of MAYV have not been reported, sporadic cases of MAYV infection have been detected.^34^–^36^ It is possible, therefore, that a case of MAYV infection could be misidentified as a CHIKV infection using the CHIK diagnostic kits without confirmation by PRNT. ONNV is closely related to CHIKV and cross-reactivity of antibodies is well described.^37^,^38^ A case of ONNV infection would likely have a positive result in the CHIK diagnostic kits.

There were limitations to the evaluations. At CDC the CHIKV-specific real-time RT-PCR is the first test for samples collected with the first 6 days of infection. Considered a confirmatory assay, no samples with positive real-time RT-PCR results were subsequently tested by MAC-ELISA. The CARPHA samples were tested at CDC using this testing algorithm. Similarly, because information on timing of sample collection was not provided for most samples received at NML, samples were first tested by MAC-ELISA, and only those with positive results were tested by real-time RT-PCR. No samples with negative CHIKV MAC-ELISA results were tested by real-time RT-PCR. Therefore, with these sample sets there was no way to assess if any of the commercial IgM detection assays had higher CHIKV IgM sensitivity than that of the CDC or NML CHIKV MAC-ELISA, as the panel did not include samples with positive real-time RT-PCR and negative CDC or NML MAC-ELISA results. The limited sample sets of each laboratory in this study illustrate the importance of establishing and sustaining biobanks for assay assessments for CHIKV and other rare and emerging viruses.

In summary, the Euroimmun Anti-CHIKV ELISA (IgM), InBios CHIKjj *Detect* MAC-ELISA, Abcam Anti-CHIKV IgM human ELISA (with CHIKV-specific IgM serum controls), and Euroimmun Anti-CHIKV IIFT kits were shown to have equivalent performance to the reference assays by which they were evaluated. These commercially available, validated kits provide laboratories with multiple options for serological testing for CHIKV infections. Laboratories should implement QC with an IHPC to ensure the reliability of kit performance. Any modifications to the kits by the manufacturers will necessitate reevaluation.

## Figures and Tables

**Table 1:** Characteristics of nine commercial CHIK IgM detection assays

Manufacturer	Assay name and format	No. of samples per kit	Sample volume (μL)	Estimated time to test 20 samples (hours)	Storage conditions (°C)	Evaluated by laboratory
Microplate MAC-ELISA						
Abcam (Cambridge, UK)	Anti-CHIKV IgM human ELISA	91	10	4	2–8	CARPHA, CDC
CTK Biotech (San Diego, CA)	Recombilisa CHIK IgM Test	91	10	2	2–8	CDC
Euroimmun (Luebeck, Germany)	Anti-CHIKV ELISA (IgM)	93	2	3.5	2–8	CARPHA, CDC, and NML
Genway (San Diego, CA)	CHIKV IgM μ-capture ELISA	91	10	4	2–8	CDC
InBios (Seattle, WA)	CHIKjj *Detect* MAC-ELISA	92	4	3.5	Most 2–8; antigen −20 to −80	CARPHA, CDC
SD Standard Diagnostics (Yongin-si, Gyeonggi-do, Republic of Korea)	CHIKa IgM ELISA	91	10	2	2–8	CDC
Rapid test						
CTK Biotech	*On-site* CHIK IgM Combo Rapid test	30	30	0.5	2–30	CDC
SD Diagnostics	SD BIOLINE Chikungunya IgM	25	50	0.5	1–30	CDC
Indirect immunofluorescence assay						
Euroimmun	Anti-CHIKV IIFT (IgM)*	50	∼15	3	2–8	CARPHA, CDC

CARPHA = Caribbean Public Health Agency; CDC = Centers for Disease Control and Prevention; CHIKV = chikungunya virus; IIFT = indirect immunofluorescent test; MAC-ELISA = IgM antibody-capture enzyme-linked immunosorbent assay; NML = the Public Health Agency of Canada National Microbiology Laboratory.

* Immunosorb IgG depletion sample buffer purchased separately.

**Table 2:** Serological and travel history characteristics of serum samples used for Euroimmun IgM ELISA kit evaluation at the NML (*N* = 247)

	CHIKV IgM+*	CHIKV IgM−†
Number of samples tested	100	147
Serology		
Confirmed by PRNT only	54	0
Confirmed by real-time RT-PCR only	10	0
Confirmed by HAI only	13	0
Confirmed by both PRNT and HAI	17	0
Confirmed by both real-time RT-PCR and HAI	3	0
Confirmed by both PRNT and real-time RT-PCR	2	0
Confirmed by PRNT, real-time RT-PCR, and HAI	1	0
Travel history		
Travel to Americas‡	63	31
Travel to Asia	2	4
Travel to Africa	2	1
Travel to Oceania	2	0
No travel history provided	31	111

CHIKV = chikungunya virus; ELISA = enzyme-linked immunosorbent assay; HAI = hemagglutination inhibition assay; PRNT = plaque reduction neutralization assay; NML = the Public Health Agency of Canada National Microbiology Laboratory; RT-PCR = reverse transcription polymerase chain reaction.

* Serum samples were classified as originating from confirmed cases of CHIKV infection if CHIK IgM+ and either PRNT and/or real-time RT-PCR and/or HAI positive. Samples were identified as either CHIKV IgM+ or IgM− using an in-house CDC-based IgM ELISA.

† See Table 3 for description of sample panel with no detectable CHIK IgM antibodies.

‡ South America, Central America, or the Caribbean; two CHIKV IgM samples with travel history to Mexico were also included.

**Table 3:** Serum sample panel used at NML for determining specificity of the Euroimmun IgM ELISA kit (*N* = 147)

Pathogen-specific antibody detected (IgM and/or IgG)	No. tested by Euroimmun IgM ELISA (*N* = 147)
Cytomegalovirus	3
Dengue virus	10
Epstein–Barr virus	3
Hepatitis B virus	3
Hepatitis C virus	3
Herpes simplex virus	3
Human immunodeficiency virus	3
Japanese encephalitis virus	3
Jamestown Canyon virus	3
Parvovirus	13
Ross River virus	3
Varicella zoster virus	3
West Nile virus	4
Yellow fever virus	3
*Anaplasma phagocytophilum*	25
*Borrelia burgdorferi*	2
*Helicobacter pylori*	5
*Salmonella typhi*	1
*Treponema pallidum*	3
Mycoplasma	3
No pathogen-specific antibody detected*	48

ELISA = enzyme-linked immunosorbent assay; NML = the Public Health Agency of Canada National Microbiology Laboratory.

* Serum samples for which there were no recorded positive results for any specific antibodies to other agents.

**Table 4:** Summary results of nine CHIKV IgM detection assays evaluated at CDC

Test	Kit result	CDC CHIKV IgM+*	CDC CHIKV IgM−†	Total
CTK Recombilisa CHIK IgM Test	+	2	0	2
	−	14	4	18
	Total	16	4	20
Genway CHIKV IgM μ-capture ELISA	+	0	0	0
	−	27	16	43
	Total	27	16	43
SD Diagnostics CHIKa IgM ELISA	+	12	2	14
	−	19	11	30
	Total	31	13	44
Abcam Anti-CHIKV IgM human ELISA (30 minutes 25°C incubation), lot no. GR166537-2	+	44	2	46
	−	3	37	40
	EQ	1	3	4
	Total	48	42	90
Abcam ELISA (ON 4°C incubation), lot no. GR166537-2	+	47	1	48
	−	1	40	41
	EQ	0	1	1
	Total	48	42	90
Abcam ELISA (30 minutes 25°C incubation), lot no. GR232915-1	+	36	1	37
	−	0	32	32
	EQ	0	1	1
	Total	36	34	70
Euroimmun Anti-CHIKV ELISA (IgM)	+	51	1	52
	−	1	38	39
	EQ	0	1	1
	Total	52	40	92
InBios CHIKjj *Detect* MAC-ELISA	+	36	0	36
	−	0	35	35
	Total	36	35	71
CTK *On-site* CHIK IgM Combo RT	+	3	0	3
	−	20	4	24
	Total	23	4	27
SD BIOLINE Chikungunya IgM	+	0	0	0
	−	24	7	31
	Total	24	7	31
Euroimmun Anti-CHIKV IIFT (IgM)	+	34	0	34
	−	3	35	38
	EQ	0	3	3
	Total	37	38	75

CARPHA = Caribbean Public Health Agency; CDC = Centers for Disease Control and Prevention; CHIKV = chikungunya virus; EQ = equivocal; MAC-ELISA = IgM antibody capture enzyme-linked immunoassay; IIFT = indirect immunofluorescence test; ON = overnight; RT = rapid test.

* Includes Mayaro and o'nyong-nyong viruses IgM PC serum.

† Includes Venezuelan equine encephalitis and North American eastern equine encephalitis viruses IgM PC serum.

**Table 5:** Summary results of four CHIKV IgM detection assays evaluated at CARPHA

Test	Kit result	CDC CHIKV ELISA IgM+	CDC CHIKV ELISA IgM−	Total
Euroimmun ELISA	+	26	0	26
	−	0	10	10
	Total	26	10	36
Euroimmun IIFT	+	21	1	22
	−	0	11	11
	Total	21	12	33
Abcam ELISA (4°C incubation)	+	36	0	36
	−	0	10	10
	Total	36	10	46
InBios ELISA	+	27	1	28
	−	0	13	13
	Total	27	14	41

CARPHA = Caribbean Public Health Agency; CDC = Centers for Disease Control and Prevention; CHIKV = chikungunya virus; ELISA = enzyme-linked immunosorbent assay; IIFT = indirect immunofluorescent test.

**Table 6:** Summary of the Euroimmun, InBios, and Abcam CHIK MAC-ELISAs and Euroimmun IIFT evaluations at CARPHA, CDC, and NML

Reference laboratory (no. of samples tested)	% Sensitivity (95% CI)	% Specificity (95% CI)	Accuracy (95% CI)
Abcam ELISA*			
CDC (*N* = 68)	100 (88–100)	97 (82–100)	99 (91–100)
Euroimmun ELISA			
CARPHA (*N* = 36)	100 (84–100)	100 (66–100)	100 (88–100)
CDC (*N* = 68)	100 (88–100)	97 (82–100)	99 (91–100)
NML (*N* = 247)	94 (87–98)	96 (91–98)	95 (91–97)
Euroimmun IIFT			
CARPHA (*N* = 33)	100 (81–100)	92 (60–100)	97 (82–100)
CDC (*N* = 75)	92 (77–98)	100 (89–100)	96 (88–99)
InBios ELISA			
CARPHA (*N* = 41)	100 (84–100)	93 (64–100)	98 (86–100)
CDC (*N* = 68)	100 (88–100)	100 (87–100)	100 (93–100)

CARPHA = Caribbean Public Health Agency; CDC = Centers for Disease Control and Prevention; CHIK = chikungunya; CI = confidence interval; IIFT = indirect immunofluorescent test; MAC-ELISA = IgM antibody-capture enzyme-linked immunosorbent assay; NML = the Public Health Agency of Canada National Microbiology Laboratory.

* Revised kit. CARPHA evaluation of the Abcam kit not included, as the kit components were modified after the evaluation, invalidating the results.
